# Supplementary material for: Are carbon emissions trading and green financial instruments synergistic? -Comprehensive quantitative research based on content analysis
Source: PLoS One. 2024 Mar 7;19(3):e0298601. doi: 10.1371/journal.pone.0298601 (PMC10919601; doi:10.1371/journal.pone.0298601)
Supplement: S1 Data — (ZIP) [file pone.0298601.s001.zip › Data/地级市Z_F工作报告.zip!/┴╔─■/╠·┴δ╩╨/└┤╫╘╣1⁄2╓┌║┼í╛┬φ┐╦╩2╛▌═°í┐_2009.docx]

各位代表：

现在，我代表市第五届人民政府向大会报告工作，请予审议，并请市政协委员和其他列席会议的同志提出意见。

一、2008年及过去五年工作回顾

过去的一年，是铁岭发展史上不平凡的一年。我们在省委、省政府和市委的正确领导下，在市人大依法监督、市政协民主监督与大力支持下，团结和带领全市各族人民，全面贯彻党的十七大和十七届三中全会精神，坚持以邓小平理论和“三个代表”重要思想为指导，以科学发展观为统领，深入实施五大战略，全力推进双轮驱动，积极应对国内外诸多不确定因素，努力化解各种矛盾，全面完成了市五届人大五次会议确定的各项任务目标。

—— 综合经济实力跨上新台阶。预计全市地区生产总值实现500亿元，增长20%，比2003年增长1.4倍，在全省的位次由第12位提升到第11位；其中一二三产业增加值实现110亿元、255亿元和135亿元，分别增长6%、28%和12%，分别比2003年增长0.55倍、2.4倍和1倍，三次产业结构比由2003年的28.7：34.2：37.1调整到22：51：27。地区财政收入56亿元，增长28.7%，比2003年增长1.7倍。地方财政一般预算收入33亿元，增长43.6%，比2003年增长2.4倍，由第12位提升到第11位。全社会固定资产投资完成500亿元，增长62%，比2003年增长6.8倍，由第10位提升到第5位。工业增加值实现245亿元，增长29%，比2003年增长2.6倍，由第12位提升到第8位。社会消费品零售总额实现160亿元，增长21.5%，比2003年增长1.2倍。实际利用外资1.2亿美元，增长38.6%，比2003年增长178倍。外贸出口1.55亿美元，增长16.5%，比2003年增长1.7倍。城镇居民人均可支配收入10775元，增长20%，比2003年增长90%；农民人均纯收入5995元，增长18.8%，比2003年增长90%。

—— 工业化水平快速提升。工业增加值占地区生产总值的比重由2003年的29.9%提高到50%。沈铁工业走廊步入优化发展新阶段。重点园区面积累计达到76.5平方公里，承载能力进一步增强。产业集聚实现新突破。辽宁专用车生产基地7平方公里起步区基础设施基本建成，香港康利客车等20个项目开工建设。起重机械、阀门制造、有色金属、橡塑制品等产业集群进一步壮大。建成投产企业553户，实现工业增加值160亿元，实缴税金12.8亿元，安置就业8.5万人，支撑作用日益显现。科技创新年活动取得实效，实现高新技术产品产值107亿元，增长50%。节能减排力度加大，辽河污染治理成效显著，万元GDP综合能耗下降5.2%，主要污染物排放总量减少2%。

—— 城市面貌日新月异。凡河新城区建设进入战略转移新阶段。基础设施建设突飞猛进。累计完成投资102亿元，19平方公里区域基础设施全部建成。生态环境一展新姿。各项水系工程全线贯通，莲花湖湿地恢复工程顺利完成，绿化总面积552万平方米。重点工程拔地而起，建筑总面积305万平方米。金鹰大厦、技师学院等交付使用；市县市民服务中心、金融会馆等基本完工；本科大学、医学专科学校等开工建设。银州城区改造10件实事全部完成，城市功能进一步提升，被评为全国创建文明城市工作先进市。副中心城市和中心集镇建设迈出新步伐。调兵山市进入国家级园林城市行列，开原市晋升省级园林城市。城镇化率由2003年的31%提高到43%。

—— 对外开放取得丰硕成果。深入开展项目年活动，掀起了招商引资、开工建设、投产达效、技术改造四个高潮。在建项目950个，到位资金420亿元，其中亿元以上项目139个。新批外商投资企业25家。铁岭电厂二期、清河电厂“上大压小”一期、新优特彩板等217个项目建成投产。三洋重工、瑞宏管件等683个项目开工建设。总投资30亿元的煤矸石发电项目获得国家发改委核准。总投资26亿元的清河电厂“上大压小”二期工程获准开展前期工作。共实施技术改造项目389项，完成投资152亿元，陆平机器、铁新水泥、天祥钢铁等一批企业通过技术改造做大做强。

—— 农业农村经济形势喜人。县域经济发展势头强劲。开原市主要经济指标进入全省前10名，铁岭县、调兵山市进入全省财源大县行列。粮食生产喜获大丰收。总产量72.29亿斤，创历史最好水平。农业经济结构不断优化。畜牧业倍增计划和四个100万亩工程快速推进，畜牧业产值占农林牧渔业总产值的比重达到51%，保护地蔬菜、优质水稻、花生和榛子种植面积分别达到78万亩、90万亩、85万亩和82万亩。农业产业化进程明显加快。新建固定资产投资2000万元以上产业化项目35个，实现农业产业化产值330亿元，增长28.4%。农民培训转移力度加大。完成“阳光工程”和新型农民培训4万人，新增劳务输出5万人。农业科技水平进一步提高。推广农业新技术120项、农作物新品种50个。农业基础设施建设得到加强，有望再次荣获省“大禹杯”。农村交通事业发展迅速，村通油路率达到95%。新增农机动力12万千瓦，综合机械化水平提高2.6个百分点。完成造林29.6万亩，林业经济长足发展。农产品质量安全水平全面提高，新增绿色食品企业9家、农业标准化生产面积60万亩。动物卫生防疫体系进一步完善。气象服务及时有效。新农村建设试点工作扎实推进。为农民办的12件实事全部完成。

—— 现代服务业高起点发展。东北物流城一期工程52万平方米基本竣工。北方金融后台服务基地建设进展迅速。和谐之都主题公园前期准备工作已经完成。房地产业快速发展，开发面积502万平方米。传统服务业加快提升。新建“万村千乡市场工程”连锁店200个、“新网工程”连锁网点650个。地方粮油储备通过省政府验收，科技储粮工程快速推进。我市服务业发展得到省委、省政府的充分肯定，铁岭新城现代服务业基地被列为全省服务业十大重点项目之一，沈铁工业走廊服务产业带提升到全省服务业空间布局的战略高度。

—— 民生工程全面推进。筹集资金1.6亿元，开展各种就业培训52714人，安置实名制就业101875人。筹集资金13.1亿元，确保企业养老金按时足额发放。筹集资金1.7亿元，企业职工工资拖欠问题全部解决。筹集资金2.3亿元，帮助困难企业退休职工参加医疗保险，城镇居民医疗保险全面推进。筹集资金1.77亿元，89942户城乡低保对象实现了应保尽保，改造农村低保户危房1173户。筹集资金2700万元，解决城市廉租房1935户。筹集扶贫帮困资金3150.9万元，使特困群体的基本生活得到保障。筹集资金2204.5万元，提高了优抚对象的抚恤补助标准。筹集资金4495万元，确保困难群众温暖过冬。筹集资金1782万元，提高了13623名五保对象的供养标准。筹集资金1145万元，救助受灾群众12.7万人。筹集资金2616.8万元，实施农村扶贫开发项目133项。筹集资金3000万元，建设农村饮水安全工程249处，受益人口20.1万人。累计筹集资金1.6亿元，西丰诚信水库胜利竣工并开始向城区供水。

—— 社会事业成就斐然。教育年活动取得新成果。共筹集资金4.87亿元，改造农村中小学D级危房5万平方米，新建农村九年一贯制学校12所，义务教育两类新三片“普九”提高水平达标通过省政府验收。筹集捐资助教款物1000万元。外事学校、工程技术学校顺利通过国家级重点职业学校验收。银州区社区建设进入全省先进行列，社区卫生服务体系建设经验在全国推广。新农合筹资标准由人均50元提高到100元，乡村两级卫生机构基础设施建设成效显著。文化事业更加繁荣。工笔画、手指画画展在上海成功举办，认真开展了第三次文物普查工作，举办了第三届广场文化活动和小戏、小品、二人转大赛。广播电视基础设施更加完善，数字化平移工作走在全省前列，网络整合产业化实现了历史性突破。群众体育蓬勃开展，竞技体育再传捷报。低生育水平继续稳定，人口出生率和自然增长率分别控制在5.18‰和1.58‰。

—— 体制机制创新实现重大突破。投融资工作开创新局面。创造性地发行了6亿元的“2008铁岭投资债”。城市经营性用地全部实行市场化运作，获取土地出让金9.6亿元。市商业银行体制改革基本完成，存款突破100亿元大关。全省首家村镇银行新星村镇银行正式挂牌运营。凡河新城区融资工作成为体制机制创新的成功范例。坚持政府主导、市场运作，搭建多个融资平台，开辟广阔的融资渠道，保证了新城区建设资金需求。组建新城区建设管理委员会，探索市县联动建设管理城市的新模式。事业单位、行政管理体制、国有工业企业、粮食企业、林权制度、农垦企业等项改革也都取得了新进展。

—— 社会总体保持和谐稳定。切实加大了信访和市民投诉工作力度，妥善处理了一批历史积案。安全生产形势总体平稳，呈现事故起数、死亡人数、直接经济损失“三下降”趋势。建立了覆盖城乡的食品安全监管体系，确保了食品卫生安全。认真贯彻实施《突发事件应对法》，应急管理工作得到规范。出色完成奥运安保任务。成功破获“辽宁二号”公案。构建了城乡治安科技防范新机制，组织开展了人民调节主题实践活动，平安建设扎实推进，成为全省最稳定地区之一。依法行政制度体系更加健全，政府法制监督得到加强，依法行政水平明显提高。民族关系和谐融洽，宗教事务管理走上法制化、规范化轨道。廉政建设切实加强，经济发展环境进一步优化。全年办理人大代表建议和政协委员提案449件，代表和委员满意率达到98%以上。

外事、侨务、台办、统计、审计、物价、综合执法、档案、国税、地税、电业、规划、质量技术监督、出入境检验检疫、烟草、人防、国家安全、武警消防、新闻出版、地震、政务接待、机关事务管理、科协、残联、金融保险、政府采购、邮政、通信、驻外联络、市志、老龄、仲裁等工作也都取得了新成绩。

各位代表，随着2008年各项任务的完成，本届政府任期即将结束。过去的五年，勤劳智慧的铁岭人勇于变革，大胆创新，探索出一条经济欠发达地区超常规跨越式发展的成功之路，创造了一个践行科学发展观的新模式。过去的五年，铁岭发生了沧海桑田般的变化，所有变化集中到一点，就是建设了沈铁工业走廊，结束了传统农业地区的历史，开辟了工业化的新纪元。过去的五年，铁岭创造了一个又一个奇迹，其中最大的奇迹就是规划建设凡河新城区，加速了城镇化进程，走进了城镇化的新时代。过去的五年，铁岭经济社会建设取得了令人瞩目的成就，主要经济指标实现了几代铁岭人梦寐以求的位次前移，大踏步地赶上了时代的新潮流。在漫漫的历史长河中，五年只是一个瞬间，但过去的五年作为一个特殊的历史单元，必将彪炳史册。

五年来，我们之所以取得上述成就，最关键的是牢牢把握了以下三个基本点。一是坚持改革开放。高举改革开放大旗，积极推进各项改革，大胆创新体制机制，极大地激发了经济社会发展活力。大力开展招商引资，努力在项目建设上寻求新突破。我们连续四年开展项目年活动，在全市不断掀起招商引资、开工建设、投产达效、技术改造四个高潮，特别是在大项目建设上实现了历史性突破，为经济社会超常规跨越式发展注入了不竭动力。二是推动科学发展。针对“一小两低”的市情，全力推进双轮驱动，努力在沈铁工业走廊和凡河新城区建设上寻求新突破。沈铁工业走廊和凡河新城区建设开辟了发展空间，构筑了发展载体，提升了铁岭地位，扩大了对外影响，为经济社会超常规跨越式发展提供了强大支撑。三是促进社会和谐。牢牢把握维护稳定大局，努力在改善民生上寻求新突破。我们始终坚持以人为本、执政为民，从就业、社保、医疗、住房等老百姓关心的热点难点问题入手，加大民生工程实施力度，让改革发展成果更多地惠及百姓，以民生工程促稳定、促和谐，为经济社会超常规跨越式发展奠定了坚实基础。

各位代表，万丈高楼平地起。今天的成就，凝聚着历届政府的不懈努力，饱含着全市人民的辛勤汗水。在此，我代表市第五届人民政府，向奋战在各条战线上的全市各族人民，向一直积极支持政府工作的市人大代表、市政协委员、各民主党派、工商联、无党派人士、人民团体、中省直驻铁单位及社会各界人士，向解放军驻铁部队指战员和武警官兵，向所有关心、支持、参与新铁岭大铁岭建设的广大客商和海内外朋友表示衷心的感谢并致以崇高的敬意！

在看到成绩的同时，我们也要清楚地看到目前存在的主要矛盾和问题。一是经济总量小的局面还没有根本改变，经济发展方式还比较粗放，经济结构不尽合理，自主创新能力不强，节能减排和环境保护压力很大。二是由于区位条件、产业基础等原因，区域经济发展不平衡问题十分突出，制约贫困地区发展的深层次矛盾仍然很多。三是人民群众收入水平特别是城镇居民人均可支配收入还比较低，全市城乡还有相当数量的困难群体。四是以城带乡、以工促农的能力较弱，农民持续增收难度加大，农村各项社会事业发展相对滞后。五是政府职能转变还不适应新形势、新任务的要求，软环境还有不尽人意之处。这些问题已经引起我们的高度重视，并将采取有力措施继续认真加以解决。

二、今后五年的奋斗目标和战略重点

今后五年，是全面建设小康社会的关键时期。当前，我市总体上已经进入工业化、城镇化的重要阶段，进入加快转变经济发展方式、推动产业结构优化升级的关键时刻，进入破除城乡二元结构、加快城乡经济社会发展一体化进程的重要时期。我们要准确把握当前发展的阶段性特征，深刻认识工业化、城镇化面临的新形势、新任务，科学分析在世界金融危机中面临的新机遇、新挑战，着力解决在建设沈阳经济区和突破辽西北进程中面临的新课题、新矛盾，主动顺应人民群众过上美好生活的新要求、新期待，并将其作为推进改革、谋划发展的根本依据。坚持改革开放，必须抓住招商引资这个关键；推动科学发展，必须突出工业化、城镇化这个主题；促进社会和谐，必须关注改善民生这个大局；抓住和用好扩大内需、促进增长的重要战略机遇期，必须围绕项目年活动这个重点。

今后五年，政府工作的指导思想是：全面贯彻落实党的十七大和十七届三中全会精神，坚持以邓小平理论和“三个代表”重要思想为指导，以科学发展观为统领，深入实施五大战略，全力推进双轮驱动，加快转变经济发展方式，着力调整优化经济结构，进一步扩大改革开放，更加注重改善民生和社会建设，努力实现工业小市向工业大市的跨越、农业大市向农业强市的跨越、中等城市向大型城市的跨越、经济总量全省排名第三集团向第二集团的跨越，全面实现新铁岭大铁岭建设的宏伟目标。

今后五年，全市经济和社会发展的奋斗目标是：

—— 综合经济实力实现明显位次前移。地区生产总值年均增长17%以上，到2013年实现1200亿元。

——经济运行质量和效益大幅提升。地区财政收入年均增长18%以上，到2013年实现130亿元。地方财政一般预算收入年均增长22%以上，到2013年实现90亿元。城镇居民人均可支配收入年均增长18%，到2013年达到2.5万元。农民人均纯收入年均增长12%，到2013年突破1万元。

—— 城镇化水平再上新台阶。全市城镇人口160万人，城镇化率52%。凡河新城区19平方公里起步区基本建成，银州城区功能不断提升，中心城区人口争取达到100万人，生态型、数字化城市基本建成。

今后五年，全市经济社会发展的战略重点是：

1．以建设凡河经济先导区为重点，优化经济布局。紧紧抓住建设沈阳经济区和突破辽西北双重战略机遇，顺应区域经济发展客观规律，根据不同区域资源环境承载能力、发展基础和比较优势，将全市划分为两大经济板块，即以凡河新城区为中心的凡河经济先导区和以昌图、西丰为重点的县域经济突破区。通过寻找突破口，优先发展凡河经济先导区，集聚能量，产生扩散效应，辐射和带动昌图、西丰跨越式发展，最终实现区域经济协调可持续发展。凡河经济先导区以凡河新城区为中心，以银州区、铁岭县、开原市、调兵山市、清河区为腹地，以辽宁专用车生产基地、开发区和高新区为支撑，通过城市规划、基础设施、产业布局、生态环境、政策措施五位一体的制度安排，实现空间布局同城化，经济社会一体化，要素无缝链接，产业错位发展，把凡河新城区建成经济高速发展、社会繁荣昌盛、人民安居乐业、生态良性循环的区域性中心城市；把凡河经济先导区建成中心隆起、轴线辐射、城乡一体、组团发展的城市群、产业带、经济圈。凡河经济区要率先发展，将其建成改革开放的先导区、科学发展的示范区，率先实现全面小康，率先基本实现现代化。未来的凡河经济区既是沈阳、大连北向发展的纵深腹地，又是长春、哈尔滨南向入海的桥头堡，承南启北，位居要津，成为东北地区重要的经济增长极。昌图、西丰是全市区域经济的重要组成部分，要继续举全市之力，在政策、资金、物资等方面予以倾斜，使其成为县域经济突破区，将其建成全国最大的换热设备产业基地、鹿产品精深加工基地、农副产品精深加工基地、新型能源基地和重要的商品粮基地。加强基础设施建设，加快社会事业发展，早日脱贫致富，走向全面繁荣，实现在全省位次前移。

2．以转变经济发展方式为重点，推进产业结构优化升级。专用车生产基地和换热设备产业基地全部建成，专用车生产基地引进生产企业100家，实现产值1000亿元，把铁岭建成中国一流、世界先进的专用车生产基地。换热设备产业基地入驻企业160家以上，实现产值300亿元。加快发展新型能源工业，完成铁岭、清河两大电厂技术改造，风力发电、煤矸石发电、生物质发电等项目全部建成，装机总容量突破800万千瓦，大强煤矿、昌图亮中煤矿建成投产。全力打造农产品精深加工基地。扩大新型建材业生产能力，新型干法水泥年产突破1000万吨。继续培育壮大装备制造、有色金属、起重机械、橡塑制品等产业集群。加快建设以东北物流城为重点的铁岭新城现代服务业基地和沈铁工业走廊服务产业带，大力发展现代服务业。以引进高科技项目为突破口，深入开展科技创新年活动，为经济社会发展提供强力支撑。到2013年，三次产业结构比调整到15：50：35。

3．以深化农村改革为重点，加快建设社会主义新农村。全面推进农村各项改革，建立和完善农村基本经营、土地管理、农业支持保护、现代农村金融、城乡经济社会发展一体化和民主管理等项制度。加强农业物质技术装备，大力调整优化农业结构，加强农业基础设施建设，建立新型农村社会化服务体系，提高农业标准化和农产品质量安全水平，实施百亿斤粮食工程，提高农业的综合生产能力和抗风险能力。扩大公共财政覆盖农村的范围，积极发展农村文化、教育、医疗卫生等公共事业，建立健全农村社会保障体系和社会管理体系，逐步形成城乡经济社会发展一体化新格局。到2013年，粮食总产量达到80亿斤；农业增加值实现180亿元，年均增长7%。开原市进入全国百强县，铁岭县进入全省前10名。

4．以节能减排和环境保护为重点，建设资源节约型、环境友好型社会。在全市重点企业推行清洁生产，大力发展环保产业和循环经济，淘汰落后产能，加强以绿化为重点的生态建设。万元GDP能耗降低20%，化学需氧量排放减少8%，二氧化硫排放量减少7%，城市空气优良天数稳定在330天以上。

5．以民生工程为重点，努力构建和谐铁岭。加快完善就业、社会保障、增加城乡居民收入、最低生活保障、保障性安居工程、教育、卫生、文化、人居环境、平安建设等“十大民生体系”，努力使全体人民学有所教、劳有所得、病有所医、老有所养、住有所居。切实加强信访、安全生产、应急管理、社会治安防控等社会管理工作，确保社会和谐稳定。

三、2009年的主要工作

2009年是新一届政府的开局之年，做好今年的工作，具有十分重要的意义。当前，我们既面临国际金融危机影响持续加深的严峻挑战，更面临国家扩大内需、促进增长的良好机遇。我们要审时度势，坚定信心，变压力为动力，化挑战为机遇，确保主要经济指标增幅继续走在全省前列，为完成未来5年的奋斗目标奠定坚实基础。

主要预期目标是：地区生产总值600亿元，增长18%。地区财政收入68亿元，增长17%。地方财政一般预算收入40亿元，增长21%。全社会固定资产投资650亿元，增长30%。规模以上工业增加值实现300亿元，增长23%。社会消费品零售总额192亿元，增长20%。实际利用外资1.44亿美元，增长20%。外贸出口1.86亿美元，增长20%。城镇居民人均可支配收入1.28万元，增长18.8%。农民人均纯收入6720元，增长12%。城镇登记失业率控制在3.8%以下，人口自然增长率控制在4‰以内。

为实现上述目标，着力抓好以下9项重点工作：

（一）抢抓国家扩大内需机遇，继续掀起项目年活动新高潮

积极向上争取项目和资金。把抢抓机遇落实到具体的项目上，只争朝夕地做好434个项目的各项基础工作。对事关全局的沈铁城际轻轨、东北物流城二期等55个重大项目，继续实行领导包扶责任制，确保取得实质性进展。全力推进100个亿元以上重点项目建设，确保煤矸石发电等50个在建项目建成投产，中利电缆等50个项目基本建成。争取铁煤集团60万千瓦热电联产项目开工建设。继续加大招商引资力度。精心组织好长三角、珠三角、香港等境内外招商活动，深入开展沈阳招商月活动，确保引进域外资金500亿元，亿元以上项目200个。继续推进“1123”工程。实施技术改造项目400项，完成投资200亿元。

（二）采取积极应对措施，保持工业经济强劲增长势头

进一步优化沈铁工业走廊，科学地确定重点工业园区的产业定位和功能分区，实现主业突出、优势互补、配置合理、错位发展。进一步完善园区功能。完成基础设施投资15亿元，新增园区面积15平方公里。完成园区总体环境评价，加快污水处理厂建设，入驻企业全部实现达标排放。继续完善一门代办受理、一次会议审批、一个公章生效模式，不断提升服务水平。加快做大产业集群。专用车生产基地引进企业70户，开工建设企业50户，投产企业30户。换热设备产业基地起步区基本建成，引进企业50户，开工建设企业30户。起重机械、有色金属、阀门制造、塑料模具、橡塑制品等生产基地进一步扩大规模，提高档次。为工业发展营造良好环境。积极打造融资平台，拓宽融资渠道，推进银企对接，组建市县担保公司，切实解决企业融资难问题。加快推进新型工业化进程。继续开展科技创新年活动，新增高新技术企业40户,实现高新技术产品产值160亿元，增长50%。对铁煤集团热电厂、开原宏达热电公司等企业实施脱硫治理，建成7座城市污水处理厂，对污染严重的企业坚决予以关停。万元GDP能耗下降5%，化学需氧量排放量下降3.5%，二氧化硫排放量下降2.6%。

（三）全力推进凡河新城区建设，继续改造提升银州城区功能

凡河新城区建设要加快实施“四个战略转移”：即由基础设施建设向城市建设转移，由政府投入向市场投入转移，由建设城市向经营城市转移，由单一建设向建设与管理并重转移。努力在重大项目、招商引资、土地出让、人口集聚、资产整合上实现五个新突破。今年要完成投资60亿元，新开工建设面积300万平方米。重点实施10项工程：⒈沈铁城际轻轨、凡河新区至调兵山快速通道等对外交通工程。⒉热电联产、城市污水处理、道路桥梁等基础设施完善工程。⒊莲花湖湿地恢复、凡河治理、街路绿化、园林景观等城市生态建设工程。⒋五星级酒店、商业广场等现代服务业工程。⒌本科大学、城乡一体化中学等公共事业工程。⒍天水河两侧区域开发等商住工程。⒎金融后台服务基地建设工程。⒏辽宁专用车生产基地建设工程。⒐东北物流城建设工程。⒑和谐之都主题公园建设工程。继续改造提升银州城区功能，重点抓好11件实事：⒈争创国家级园林城市。⒉启动铁西热电联产项目。⒊改造铁西南出口和东北出口。⒋继续实施自来水二次加压设备改造。⒌建设10座地坑式垃圾中转站和垃圾处理场渗沥液工程。⒍继续实施小街小巷环境综合整治。⒎普及垃圾收集袋装化。⒏改造城南排水系统。⒐新建和改造污水泵站。⒑改造铁路天桥。⒒提高居民小区物业管理覆盖率。继续抓好副中心城市和20个中心集镇建设。

（四）大力发展现代农业，开创新农村建设新局面

努力提高粮食综合生产能力。启动百亿斤粮食工程，稳定粮食产量。大力调整优化农业结构。深入实施畜牧业倍增计划和四个100万亩工程，新增标准化畜牧小区195个，实现畜牧业产值154亿元，增长15%。加强动物卫生防疫，确保不发生大的疫情。新开发保护地蔬菜、优质水稻、花生、榛子39万亩。完成人工造林25万亩，实现林业产值45亿元。全面提升农业产业化经营水平。进一步加大对嬴德、大成、雨润、宏福、大牛、德冠等20户重点龙头企业的扶持力度，新增固定资产投资2000万元以上产业化项目20个，农业产业化产值实现400亿元，增长15%。加强各类种养基地建设，推进西丰县养鹿业规模化经营。切实加强农业标准化和农产品质量安全建设。建设1个市级农产品检测中心、7个县级检测站，推广测土配方施肥820万亩，完成生物防治面积850万亩，新增绿色食品标识15个、无公害农产品认证30个、农业标准化生产面积50万亩。加强农业基础设施建设。完成农建土石方3000万立方米，建设骨干工程200项，继续争夺省“大禹杯”。完成10座病险水库除险加固。建设年处理畜禽粪便6万吨的大型沼气池及生物质发电项目，受益农户4.2万户。深化农村改革。在依法、自愿、有偿的基础上，积极推进土地承包经营权流转和土地股份合作经营。加快发展以农民专业合作社为重点的农村各类专业合作组织，进一步提高农民的组织化程度，农民专业合作社达到550家以上。围绕广大农民的民生问题，着力办好14件实事：1.新建饮水安全工程200处，解决20万人口饮水安全问题。2.实施开发式扶贫工程，实现40个村、5万人脱贫。3.改造特困户D级危房700户。4.新建清洁能源工程15处，新发展沼气用户2000户。5.确定185个新型农民培训示范村，培训转移富余劳动力5万人。6.完善农村最低生活保障制度，确保4.5万户、8.8万名低保对象应保尽保并逐步提高保障水平。7.创建省级环境优美乡镇6个、村屯50个。8.新建村级农家店100个。9.新建乡镇综合文化站20个、农家书屋200个。10.新建体育健身休闲广场80个。11.新建九年一贯制学校4所，改造中小学D级危房3万平方米。12.新建计划生育中心服务站9所。13.创建省级卫生村10个，市级卫生村20个。14.新建、改扩建5所中心敬老院。

（五）加快发展服务业，培育新的经济增长点

全力打造铁岭新城现代服务业基地。加快东北物流城二期工程建设，确保小商品市场和东北机动车交易市场开业。和谐之都主题公园和湿地博物馆全面开工建设。落实“国民休闲计划”，组织好特色乡村游活动。加快北方金融后台服务基地建设，积极推进中国建行等单位金融后援项目建设。装备制造学校、本科大学、体校、军体校、铁岭医专等5所院校竣工并投入使用；开工建设装备制造学院、铁岭县职教中心、职业教育实训基地，职业院校在校生人数达到1.5万人。规划建设沈铁工业走廊服务产业带。重点培育开原工业物流、调兵山生产性服务、西丰鹿产业、昌图农产品专业市场四大产业集群，抓好开清民间艺术文化、西丰自然生态、调兵山工业观光三大旅游产业带建设。促进房地产业又好又快发展。制定出台扶持房地产业发展的优惠政策，降低房地产交易成本，努力搞活房地产市场，发挥房地产业在扩大内需中的积极作用。浅水湾一号全部竣工并交付使用。全市新开发房地产780万平方米，完成投资100亿元，实现产值150亿元。加速提升传统服务业。新发展规模以上连锁店15个、“万村千乡市场工程”农村连锁店100个、农村社区服务中心5个。全面落实粮食储备任务，向农村推广新型储粮仓1.5万套。

（六）认真落实财税金融政策，为经济社会又好又快发展提供保障

继续加大融资力度。进一步加强与国家开发银行、农发行和国有商业银行的合作，积极推进信托融资项目，完善融资平台，创新融资方式。积极向上争取资金。充分发挥争取资金平台和项目源管理系统的作用，尽最大可能争取上级专项资金。充分发挥金融对经济发展的支撑作用。认真落实适度宽松的货币政策，积极支持国有商业银行全面恢复贷款权限。制定优惠政策，鼓励金融机构多投放，加大对地方经济发展的信贷支持力度。加强金融生态环境建设，努力打造诚信铁岭。组建开原市村镇银行，力争引进2家金融投资机构。继续做大财政“蛋糕”。加强税收征管，杜绝跑冒滴漏，做到应收尽收。继续深化部门预算改革，完善国库集中支付制度，积极推行乡财县管。牢固树立过紧日子思想，大力开展增收节支。优化财政支出结构，切实增加对“三农”、就业、社会保障、教育、医疗、中小企业等方面的投入。

（七）加大民生工程建设力度，努力构建和谐铁岭

千方百计稳定和扩大就业。以困难群体、高校毕业生和农民工为重点，深入实施全民创业促就业工程，加快建设城乡统一的劳动力市场，加大普惠制就业培训力度，开展有组织的劳务输出，全年安置实名制就业5万人，确保零就业家庭至少有一人稳定就业。广开农民工就业门路，扶持有能力、有条件的农民工返乡创业，加大劳动保障执法监察力度，通过建立保证金等措施确保农民工工资足额发放。不断完善社保体系。进一步健全以“五险合一”为重点的社会保险制度，扩大保险覆盖面，认真解决进城务工人员和失地农民的社会保障问题。继续完善城乡最低生活保障制度，努力做到应保尽保，并积极创造条件提高保障标准。加强社会救助和社会福利工作，建设和完善8所社区服务中心。建设城市保障性安居工程50万平方米，改善1万户困难群众的居住条件。切实加强社会管理，维护社会稳定。深入开展解难题促和谐活动，集中解决信访突出问题。延伸市长公开电话触角，畅通利益诉求渠道，千方百计为人民群众排忧解难。进一步完善应急管理体制，妥善处置各类突发事件。落实企业安全生产主体责任，开展企业安全生产标准化创建活动，强化安全生产监管，坚决遏制各类重特大安全生产事故。建立严格的食品药品生产标准，严厉打击各种违法违规生产经营行为，确保人民群众生命健康安全。深入开展普法活动，及时排查调处社会矛盾，加快警务信息化建设，提高社会治安防控能力，努力打造平安铁岭。

（八）积极发展各项社会事业，切实加强精神文明建设

扎实开展教育年活动。深入实施素质教育，进一步改善办学条件，创建省级标准化九年一贯制学校5所。在凡河新城区新建标准化示范高中1所、初中2所。启动新一轮义务教育“两类新三片”双高水平普九达标工作。继续提高高中教育教学质量。大力发展职业教育，争创省级品牌专业2个。本科大学、医学专科学校申报成功，新校址交付使用。继续开展捐资助教活动，募集教育基金1000万元。强化卫生服务体系建设。巩固农村三级医疗卫生服务网络，完善新型农村合作医疗制度，提升城市社区卫生服务功能，为城乡居民提供质优价廉、方便快捷的医疗卫生服务。加强公共卫生服务体系建设，提高重大疫病防控和突发公共卫生事件处置能力。继续稳定低生育水平，有效治理出生人口性别比偏高问题。扎实推进文化名城建设。在新城区筹备建设文化艺术博览中心。做好银冈书院、西丰城子山国家级文物保护单位申报工作，开展第三次文物普查。组织好新农村、和谐社区文艺展演活动，丰富城乡文化生活。继续巩固、扎实推进广播电视村村通工程，全面完成县（市）区城区有线广播电视数字化整体平移，积极发展文化传媒产业，提升文化软实力。高度重视网络媒体作用，正确引导社会舆论。广泛开展体育运动。坚持开展全民健身活动，深入实施百万农民健身工程，农村村屯、城市社区体育设施覆盖率分别达到30%和50%以上。认真组织参加各项体育赛事，争取再创佳绩。切实加强精神文明建设。广泛开展文明单位、文明城市、文明乡村创建活动，继续开展窗口行业选优评差，培育文明和谐的良好社会风尚，争创全国文明城市，为经济社会发展提供思想保证、舆论支持和文化条件。

（九）切实加强政府自身建设，为实现各项任务目标提供坚强保证

各位代表，本次大会将选举产生新一届市政府领导班子。新一届政府面临着全面实现新铁岭大铁岭建设目标的繁重任务。搞好政府自身建设，是全市人民的期望，更是实现各项奋斗目标的保证。为此，新一届政府要认真开展科学发展观学习实践活动，按照务实、高效、廉洁的要求，全面开创政府自身建设的新局面。加强民主政治建设。认真执行人大及其常委会的各项决议、决定，自觉接受人大的依法监督和政协的民主监督，认真办理人大代表建议和政协委员提案，加强与民主党派、工商联、无党派人士、人民团体和社会各界人士的联系，广泛征求专家学者和人民群众的意见建议，努力做到民主决策、科学决策。加强政府法制建设。建立学法用法制度，切实提高广大公务员特别是领导干部依法行政意识。深化行政执法体制改革，逐步实现执法重心下移。全面落实行政执法责任制、评议考核制和责任追究制，加强行政复议和应诉工作，进一步完善监督制约机制。加强软环境建设。深入扎实地开展软环境建设年活动，切实解决人民群众和企业反映强烈的增加企业负担、行政效能低下等问题。深化行政审批制度改革，继续向县（市）区下放权力。积极探索公共行政服务网络向基层延伸，逐步建立市县乡村四位一体、上下联动的公共行政服务新机制。认真落实限时办结、超时默认等制度，加强电子政务建设，努力提高办事效率。加强机关作风建设。深入实际，深入基层，深入群众，认真开展调查研究，做到问政于民，问需于民，问计于民。加强政务督查，狠抓工作落实。认真开展民主评议行风活动，并将评议结果作为政府部门选优评差的重要依据。加强政府廉政建设。全面推进政务公开，扩大行政权力公开透明运行试点，从源头上遏制腐败。严肃查处各种违法违纪案件，对典型案件公开曝光，努力建设廉洁政府。

各位代表，回首过去，我们携手创造了奋进崛起的辉煌历史；展望未来，我们共同肩负着跨越腾飞的神圣使命。让我们更加紧密地团结在以胡锦涛同志为总书记的党中央周围，高举中国特色社会主义伟大旗帜，在中共铁岭市委的正确领导下，励精图治，继往开来，为全面实现新铁岭大铁岭建设宏伟目标而努力奋斗！
